# Precision oncology without borders: the role of biobanking in integrating Africa’s genetic diversity into global genomic research

**DOI:** 10.1038/s41698-026-01591-y

**Published:** 2026-07-09

**Authors:** Lina Winter, Haby Konaté, Fousseyni Diarra, Bakrou Kamaté, Mamoudou Maiga, Cheick B. Traoré, Sonja Loges, Frederik Marmé, Saidou Balam, Christoph Brochhausen

**Affiliations:** 1 Institute of Pathology, Medical Faculty Mannheim, Heidelberg University, Mannheim, Germany; 2 DKFZ-Hector Cancer Institute at the University Medical Center Mannheim, Mannheim, Germany; 3 Division of Rheumatology, Department of Medicine III, Medical University of Vienna, Vienna, Austria; 4 Mali International Center for Excellence in Research, University of Sciences, Techniques and Technologies of Bamako, Bamako, Mali; 5 Integrated Department of Personalized Oncology, University Hospital Mannheim, Medical Faculty Mannheim, Heidelberg University, Mannheim, Germany; 6 Department of Obstetrics and Gynecology, Medical Faculty Mannheim, Heidelberg University, University Medical Center Mannheim, Mannheim, Germany

**Keywords:** Cancer, Computational biology and bioinformatics, Genetics

## Abstract

Cancer mortality in sub-Saharan Africa is projected to double by 2030. African populations exhibit the highest genetic diversity worldwide, impacting cancer susceptibility, tumor biology, and therapeutic response, yet remain critically underrepresented in biomedical research. This narrative review examines biobanking and genomic research infrastructure in sub-Saharan Africa and its implications for precision oncology, highlighting that locally governed, sustainable biobanks and equitable international partnerships are fundamental prerequisites for *Precision Oncology Without Borders*.

## Introduction

As a young and rapidly evolving discipline, biobanking has become an indispensable element in biomedical research. Biobanks are facilities that collect, preserve, store, and disseminate biological specimens and their related data while adhering to standardized operating procedures^1^. Thereby, enormous research progress in the fields of genomics, epigenetics, personalized medicine, and epidemiology has been made possible over the last decade^2^. Despite the enormous scientific potential of biobanking, there exists a geographic gap in the availability and use of these infrastructures. For instance, high-income countries (HICs) in Europe, North America, and Asia have robust and sustainable biobank systems with local, regional and international networks^3^. In contrast, many low- and middle-income countries (LMIC) in the global South, particularly in sub-Saharan Africa (SSA), are underrepresented in the global biobanking landscape^4,5^. This imbalance reflects the socioeconomic context of many SSA nations, whose economic development has been shaped by historical factors and contemporary challenges, including governance limitations and structural inequalities^6,7^. However, since the early 2000s, many SSA countries experienced sustained economic growth, economic diversification, and increased investment in infrastructure, science, and education^8,9^. Furthermore, the continent has been undergoing a profound demographic shift. This shift is characterized by rapid population growth, urbanization, and a very young population. In 2024, approximately 18.5% of the global population lived on the African continent. A total of 60% of the population is younger than 25 years^8^. Over the past three decades, cancer incidence in SSA has approximately doubled. Without effective intervention, the *Lancet Oncology Commission* estimates that cancer mortality in SSA could nearly double to approximately one million deaths per year by 2030. Cancer incidence is projected to double again by 2040^10^. In this context, locally managed biobanks are increasingly recognized as essential research infrastructure. They support the translation of laboratory findings into diagnostics and targeted therapies that align with local needs.

The aim of the present narrative review is to describe the recent development of the biobank landscape on the African continent and its relevance for precision oncology. Furthermore, challenges in implementing sustainable biobanking structures as well as potential solutions are discussed. Given the substantial genetic, regulatory, and infrastructural heterogeneity across the continent, this review focuses primarily on SSA. North African countries have developed distinct precision medicine and biobanking initiatives^11^. We, thereby, contribute to the scientific discussion on the conditions under which *Precision Oncology Without Borders* can become an equitable reality, critically examining both the progress made and the structural barriers that remain.

## Genetic heterogeneity in African populations

The African continent is considered to be the cradle of humankind 200,000 years ago^12^. As a result of its long evolutionary history, African populations express the highest genetic diversity globally^13,14^. This genetic diversity can be explained by the so-called “Out of Africa” founder effect. Approximately 45,000 to 60,000 years ago, small groups of humans migrated out of Africa to the Eurasian continent carrying a fraction of the original gene pool; a phenomenon often referred to as genetic bottleneck^15,16^. Genomic analyses demonstrate a serial founder effect. As humans expanded geographically from Africa, each new population was formed from a smaller part of the previous population. This repeated process increased the role of genetic drift and step by step reduced genetic diversity with growing distance from Africa^16,17^. In contrast, the large and diverse populations that stayed in Africa preserved most of the original genetic diversity^16^.

Genetic diversity was shaped by evolutionary processes including mutation, selection, genetic drift, and gene flow and has important implications for adaptation, disease susceptibility, and population health^18–23^. Thus, genetic diversity has major potential for the identification of disease-relevant genetic variants and offers important insights into the molecular pathogenesis of diseases^24^. Genetic heterogeneity also leads to differences in drug response due to variations in alleles affecting drug metabolism^25,26^. For instance, a pharmacogenetic study demonstrated that the CYP2D6*17 and CYP2D6*29 alleles occur more frequently in the Ugandan population than in other populations worldwide, including other African groups. This variation affects the metabolism of multiple commonly used drugs, including tamoxifen in breast cancer therapy. Impaired *CYP2D6* function reduces the conversion of tamoxifen into its active metabolite and may lead to decreased therapeutic efficacy^27^. Therefore, genetic testing for *CYP2D6* variants would be particularly relevant in this context to guarantee safe and effective treatment. In summary, extensive genetic diversity exists not only between African and non-African populations, but also within the African continent itself. Consequently, precision medicine approaches should be based on population-specific data and avoid assuming genetic homogeneity across African populations. This genetic heterogeneity may influence future work in oncology by supporting the identification of population-specific risk variants, molecular targets, and pharmacogenomic profiles that are currently underrepresented in global datasets.

## Underrepresentation of African populations in biomedical research: implications for precision medicine

**Fig. 1:**
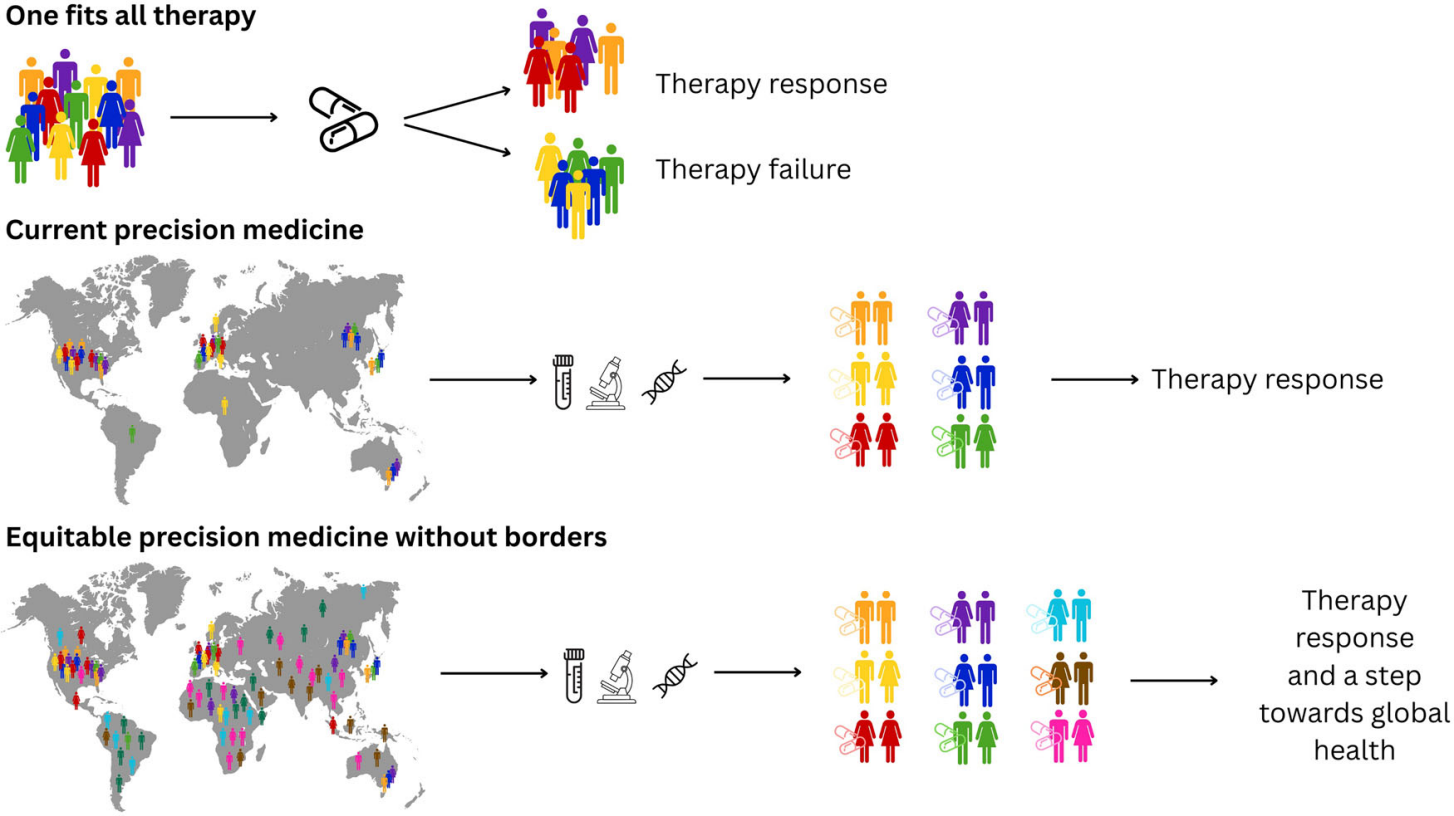
Evolution from uniform therapy to equitable precision medicine. The panel at the top illustrates the “one-fits-all” therapy approach, in which all patients receive the same treatment with variable outcomes. The middle panel shows current precision medicine, which is mainly based on genomic data from Europe, North America, and parts of Asia, resulting in limited global transferability. The bottom panel represents the ideal of equitable precision medicine without borders, integrating genetic diversity from all populations to achieve healthcare equitably efficient for all.

The limited availability of biobanks in African countries has delayed the systematic collection of biospecimens. This contributes to the underrepresentation of African populations in existing databases (Fig. 1)^28^. For instance, in The Cancer Genome Atlas (TCGA), 77% of the 5729 sequenced tumors originated from White patients, while only 12% were from Black patients. This disparity results in insufficient sample sizes to detect mutations that are moderately common (5–10%) among minority groups^29^. Correspondingly, Corpas et al. describe that individuals of European ancestry accounted for 94.48% of all samples in the genome-wide association studies (GWAS) Catalog in 2024, while those identified as “African” made up 0.19%^30^. In the PharmGKB pharmacogenomics database, a similar gap exists: 63% of samples were of European origin, compared to only 1.6% from SSA populations. Similarly, between 2015 and 2019, 76% of participants in FDA-registered clinical trials were of European descent, while just 7% were African or African American. The authors describe this imbalance as “genetic colonialism”^30^. This drastic term clearly underlines the ethical and scientific implications of systematically excluding African populations from the data that defines modern genomic medicine. In the literature, the term has been used to describe exploitative research practices involving marginalized communities, characterized by insufficient transparency, lack of benefit-sharing, and limited local governance^30^. These patterns resemble what has been described as “helicopter” or “parachute” research, where biospecimens are extracted with minimal sustained local engagement or capacity development^31^. This underrepresentation not only limits our understanding of human genetic variation. It also risks reinforcing inequities in the development of diagnostics, therapies, and risk prediction models^30^. Consequently, the identification of population-specific, targetable alterations is not given and potential clinically relevant insights in the tumor biology of African cancer patients remain unexplored. This further illustrates the complexity of implementing precision oncology in SSA in a sustainable way. In more detail, precision oncology considers genomic, environmental, and lifestyle data of patients^32^.

## Precision oncology in SSA

There are large differences in healthcare provision within SSA, which in turn has an impact on the availability of and access to precision medicine. Nations with a higher gross domestic product (GDP), such as South Africa and Nigeria have further developed health infrastructure compared to many low-resource settings^33^.

In this context, a shortage of oncologists exists throughout the region. According to a 2018 analysis, the number of practicing clinical oncologists varies markedly between countries:from 26 in Nigeria, corresponding to nearly 4000 new cancer cases per oncologist, to none in Rwanda^34^. For comparison, estimates for the countries of the European Union range between approximately 100 and 430 new cancer cases per oncologist^34^.

These findings reveal a genuine tension: if too few oncologists and researchers are available on the continent to utilize biobank specimens, the value of biobank investment may accrue primarily to external actors rather than local populations. However, this tension should not be read as an argument against biobanking investment, but rather as a call for parallel action. Biobanking infrastructure and local research capacity are mutually reinforcing. Expanded research infrastructure creates scientific career opportunities on the continent, thereby reducing the incentive for brain drain and gradually building the workforce needed to translate biobank data into locally relevant clinical insights. This lack of precision medicine is further reflected in a survey of 30 oncology centers in 21 SSA countries by Vanderpuye et al.^33^. In particular, they identified challenges in molecular characterization, access to targeted agents, imaging, and genetic risk stratification. While immunohistochemistry (ER, PR, HER2) was available in 74% of institutions, only 27% repeated testing at recurrence, as required for precision medicine. Trastuzumab was available in 66% of facilities, yet less than 20% of patients could afford a full year of therapy. Newer HER2, CDK4/6, or PARP inhibitors were practically non-existent. The authors attribute this to infrastructural and human resource limitations, as well as late presentations^33^. These findings show how limited resources currently restrict the transition from basic resources to data-driven precision medicine. Biobanking directly addresses these gaps by enabling the molecular characterization and genetic risk stratification that Vanderpuye et al. identified as lacking - provided that the resulting data remain locally governed and available for population-specific clinical translation.

## Selected cancer examples with impact on precision oncology

Patients with clearly defined genomic alterations benefit most from molecular approaches. In this context, frequent tumor entities are typically better characterized than rare malignancies due to higher case numbers and research prioritization^35^. Table 1 summarizes selected cancer entities for which population-specific genomic patterns have been described in SSA cohorts, with distinct implications for precision oncology.

**Table 1:** Selected Population-Specific Genomic Patterns Relevant for Precision Oncology in Sub-Saharan Africa

Cancer entity	Distinct molecular and genomic features reported in SSA cohorts	Potential diagnostic or therapeutic implications	Ref.
Breast cancer	Higher TNBC prevalence; elevated HRD and TMB in Nigerian TNBC and HR + /HER2- breast cancers; lower PIK3CA mutation frequency	PARP inhibitors; immune checkpoint inhibitors; lower PI3K inhibitor relevance	^33,84–88^
Prostate cancer	Gene panels derived from European ancestry (e.g. BRCA2); alternative candidate genes proposed for men of African ancestry (e.g., *PREX2*, *POLE*, *FAT1*, and *POLQ*)	DDR-targeted therapy; potential immunotherapy sensitivity	^89–92^
Colorectal cancer	Higher MSI prevalence in Nigerian cohorts, in MSS cases lower APC mutations and WNT pathway alterations, but higher RAS pathway alteration, younger age at diagnosis, increased rectal disease	Increased relevance of MSI/MMR testing; potential expansion of PD-1 inhibitor eligibility; limited benefit of fluorouracil in MSI-H tumors	^93^
Papillary Renal Cell Carcinoma	Enrichment of MET mutations in patients of African ancestry	Potential eligibility for MET inhibitors (e.g., cabozantinib, savolitinib)	^94^
Cervical cancer	HPV16/18 as predominant oncogenic types; disproportionately increased contribution of HPV35 with strong association of the HPV35 A2 sublineage with CIN3+ in women of African ancestry; enrichment of HPV clade A7 (HPV-18, HPV-45) in Ugandan cohorts with distinct epigenomic profiles and inferior prognosis compared to clade A9; host genetic variants in MHC-region loci (HLA-DRA, HLA-C, TRIM31) associated with cervical cancer susceptibility; HIV co-infection associated with younger age at diagnosis and altered tumor immune microenvironment (reduced CD4 + T cell infiltration)	Consideration of HPV35 inclusion in next-generation vaccination strategies; HPV clade-aware prognostic stratification as a potential clinical tool; consideration of HIV immune status in clinical staging and treatment planning	^95–97^
Hepatocellular carcinoma	Aflatoxin B1-associated TP53 mutations (Arg249Ser), HBV-related molecular patterns	Arg249Ser mutation serves as a region-specific diagnostic biomarker reflecting local aflatoxin exposure. Urinary metabolite panel as promising region-specific diagnostic tool. No targeted therapy currently available; standard HCC treatments	^98^

These examples show that the genomic architecture of common cancers in African populations is distinct and clinically relevant, especially in the context of the rising life expectancy and the increase of non-communicable diseases (NCD)^36,37^. Strengthening biobanking capacity is essential to detect these molecular patterns, validate biomarkers in local contexts, and extend precision medicine beyond HICs. Access should not be limited to a few research hubs or to common tumor types. Even rare cancers require attention to ensure that precision approaches become part of equitable cancer care across the continent. However, many of the therapeutic strategies outlined in Table 1 including PARP inhibitors, immune checkpoint inhibitors, and MET inhibitors remain largely inaccessible in SSA due to high costs, limited infrastructure, and restricted regulatory approval. This underlines that achieving *Precision Oncology Without Borders* requires not only population-specific genomic characterization but parallel investment in treatment access^33^.

## Development of biobanking in Africa

**Fig. 2:**
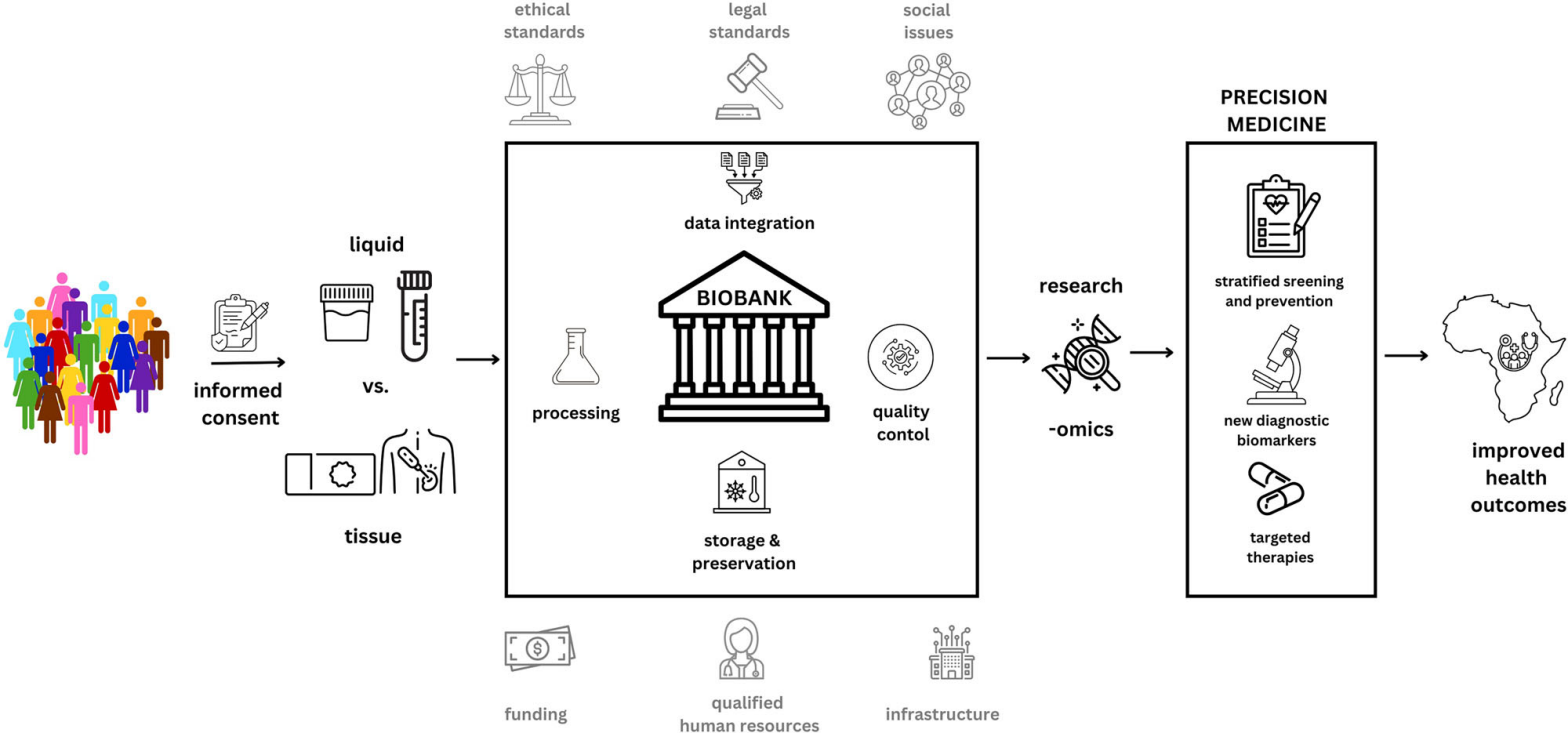
From biospecimen collection to equitable precision medicine. Schematic depiction of the function of biobanks as an important link between clinical practice and biomedical research that promote precision medicine. Biospecimen and associated data are collected, processed, and stored under standardized conditions. The light grey icons represent key framework conditions that remain limited in many African contexts, including ethical, legal, and social issues (ELSI), funding, human resources, and infrastructure. Biospecimens can be distributed to qualified research groups to support biomedical research and translational applications. Finally, this contributes to the strengthening of precision medicine.

Biobanks in general collect biological samples, such as blood and its derivatives, cells, tissue, DNA, RNA, other body fluids (urine, saliva), along with clinical and demographic data^38,39^. This allows a more precise molecular classification of patients and provides the basis for identifying and validating biomarkers. As a result, biobanks support predictive, preventive, and targeted treatment strategies, as displayed in Fig. 2^39^.

Historically, African biobanking capacities have lagged behind those of HICs, which has reinforced the underrepresentation of African population in biomedical research.

Table 2 summarizes major initiatives that have accelerated biobanking capacity building on the African continent over the past decades.

**Table 2:** Chronological Development of Major Biobanking and Genomic Capacity-Building Initiatives in Sub-Saharan Africa

Initiative	Launch Year	Geographic Scope	Primary Focus	Funding Model	Key Contribution	Sustainability	Ref.
MalariaGEN	2005	Pan-Africa & global partners	Genomic epidemiology of malaria	Wellcome Trust, Bill & Melinda Gates Foundation, NIH	World’s largest malaria genomic database; pioneer in genomic surveillance for drug and insecticide resistance; established ethical frameworks for equitable research partnerships	Ongoing consortium	^40,99^
Institut Pasteur Côte d’Ivoire / ECOWAS Regional Biobank	2009, expansion in 2018	West Africa (ECOWAS member states)	Infectious disease surveillance and epidemic preparedness	USAID (PREDICT 2), regional public funding	First regionally governed biobank network in West Africa; contributed to zoonotic pathogen surveillance across member states	Ongoing; operationally dependent on international project funding	^49^
H3Africa	2012	Pan-African (mainly SSA)	Large-scale genomic studies and standardized biorepositories across Africa	NIH, Wellcome Trust	Established pan-African genomic infrastructure; 3 regional biorepositories; massive datasets on non-communicable and infectious diseases; workforce training	Core funding ended 2023; biorepositories remain operational; selected research activities continued through successor consortium DS-I Africa	^41–43,100^
H3ABioNet	2012	Pan-African	Developed standardized bioinformatics pipelines and data analysis capacity; created a sustainable network of skilled African bioinformaticians.	NIH, Wellcome Trust	Standardized workflows; distributed bioinformatics capacity	Core funding ended 2023; transitioned into AfriGen-D	^61^
BCNet (IARC)	2013	Global incl. SSA	Biobank harmonization	WHO/ IARC	Established international quality standards and governance protocols for biobanks in low- and middle-income countries.	Ongoing network	^50,101^
ARGO	2013	Nigeria	Oncology research, biobanking, and training	NCI Center for Global Health, internal MSKCC funds, NIH	Pioneered tumor-germline profiling in Nigeria; identified unique MSI-high colorectal cancer markers in African patients; integrated biobanking into clinical care.	Active; sequencing partly external	^46^
B3Africa	2015	Africa and EU	Interoperability & IT systems	EU-funded	Development of the laboratory information system “Baobab LIMS”; BBMRI integration	Project ended 2019; Baobab LIMS remains a widely used open-source tool.	^60^
54gene	2019	Nigeria	Commercial genomic biobank	Venture capital	Large DNA collection; hospital partnerships	Collapsed in 2023 due to financial and management issues.	^53–55,102^
BioVana Research	2025	Nigeria	Genomic diagnostics	Private sector	Emerging private-sector provider with locally based precision medicine services	Very early stage	^56^

*AfriGen-D* African Genomics Data Hub, *ARGO* African Research Group for Oncology, *B3Africa* Bridging Biobanking and Biomedical Research across Europe and Africa, *BBMRI-ERIC* Biobanking and BioMolecular Resources Research Infrastructure—European Research Infrastructure Consortium, *DS-I Africa* Data Science for Health Discovery and Innovation in Africa, *EU* European Union, *H3Africa* Human Heredity and Health in Africa, *IARC* International Agency for Research on Cancer, *LIMS* Laboratory Information Management System, *MalariaGEN* Malaria Genomic Epidemiology Network, *MSKCC* Memorial Sloan Kettering Cancer Centre, *NIH* National Institutes of Health, *SSA* sub-Saharan Africa, *WHO* World Health Organization.

The chronological development of biobanking in SSA reveals a consistent pattern: infrastructure was predominantly built in response to infectious disease crises rather than in anticipation of oncological research needs. Early initiatives, such as MalariaGEN, were designed for epidemic surveillance and pathogen monitoring^40^. H3Africa, the most transformative investment in African genomic infrastructure to date, pursued a broad mandate encompassing both communicable and non-communicable diseases^41,42^. However, reflecting the historical prioritization of infectious disease research on the continent, its biorepositories - including those located in Nigeria, Uganda, and South Africa - were in practice dominated by HIV, TB, and malaria projects, with oncological research remaining the exception^43–45^. This development has direct consequences for precision oncology today: existing biorepositories, workflows, and governance frameworks were largely optimized for different specimen types, research questions, and timelines than cancer genomics requires. The African Research Group for Oncology (ARGO) represents an exception as a biobank built around oncological specimens, but it remains a single-disease, single-country initiative^46^. The field is therefore not starting from zero, but it is building on a foundation shaped by different priorities. Nonetheless, H3Africa’s genomic mandate did yield high-impact results: whole-genome sequencing of more than 400 African individuals identified over three million previously undescribed variants^47^. This underscores the scientific value of the infrastructure built and the potential that remains unrealized for oncology.

A second pattern emerging from Table 2 is the degree to which biobanking momentum in SSA has been externally driven. Current evidence suggests that, while research activity in SSA is increasing, much of the analytical capacity and agenda-setting remains externally driven, and clear indications of sustained national prioritization of biobanking and precision oncology remain limited^48^. However, there are emerging regional initiatives, such as the ECOWAS regional biobank, that indicate initial efforts toward more locally anchored governance structures^49^. Every major initiative represented in Table 2 was either initiated or predominantly funded by actors outside the continent—including the NIH, Wellcome Trust, EU, and USAID^41,42,50,51^. External funding has been crucial in building infrastructure that would not otherwise exist, but it has created a structural vulnerability. When H3Africa’s core funding ended in 2023 after approximately $180 million invested, its three regional biorepositories remained operational and key bioinformatics infrastructure transitioned into successor platforms such as AfriGen-D and DS-I Africa. However, smaller research projects struggled to complete analyses and publications without continued funding, illustrating the broader challenge of sustaining individual research capacity beyond project-based funding cycles^51^. This dynamic is compounded by chronically low domestic research investment across SSA leaving biobanking infrastructure highly exposed to shifts in donor priorities^52^. The research agenda has largely been shaped by the availability of external funding rather than by domestically defined needs, raising the question of whether “Precision Oncology Without Borders” reflects a genuinely equitable vision or risks reproducing the dependency dynamics it seeks to overcome.

A third pattern concerns the role of the private sector. The trajectory of 54gene offers the most instructive case study. Founded in 2019 with the mission to build a pan-African genomic biobank, the company achieved concrete results: it sequenced the genomes of 100,000 Nigerians across 300 ethnic groups and generated over $20 million in revenue, primarily through COVID-19 diagnostics rather than its core genomic mission^53,54^. Despite raising $45 million from prominent international investors including Y Combinator and Adjuvant Capital, the company ceased operations in 2023. Its collapse has been attributed to a combination of mission drift into COVID diagnostics, post-pandemic revenue loss, and a disputed breakdown in governance between the founder and investors that remains subject to ongoing litigation^53,55^. Two structural consequences emerge: First, genomic biobanking requires long-term capital patience that is fundamentally misaligned with standard venture capital return expectations. Second, the attempted sale of 100,000 Nigerians’ genomic data – ultimately blocked by a Nigerian federal court – illustrates both the data sovereignty risks that arise when commercial biobanking operates without adequate ethical safeguards, and the critical role of national legal frameworks in protecting population-level genomic data^53^. In summary, the collapse of 54gene reflects internal governance failures rather than an absence of market interest or scientific need for genomic biobanking in SSA. BioVana is the most recent Nigerian initiative, which underlines that demand persists. However, BioVana appears to pursue a structurally different model emphasizing local capacity building and institutional partnerships. Yet, it remains too early to evaluate its impact in this early stage^56^.

## Challenges in sub-Saharan African biobanking

The challenges faced by African biobanks and research centers are often systemic and include infrastructure, ethics, funding, and human resources. However, initiatives such as H3Africa are actively attempting to address these difficulties^51^. Table 3 provides an overview on the challenges regarding biobanking and approaches how to address them.

**Table 3:** Challenges and potential solutions in African biobanking

Category	Challenges	Potential Approaches/ Solutions
Infrastructure and Logistics	.Resource-limited environments causing inconsistent storage, unreliable power supply, and fragile cold-chain logistics^57,59^	Investment in long-term storage (−80 °C) and renewable energy backup systems; strengthening transport logistics^57,59^
	Fragmented digital and IT infrastructure, including weak internet connectivity, limited sequencing capacity, and lack of advanced data systems^52^.	Adoption of open-source management systems (e.g., Baobab LIMS)^60^, improved broadband infrastructure, hybrid cloud-based environments, and regional sequencing centers^51,52^.
Quality Management & Accreditation	Absence of standardized SOPs and limited quality accreditation^50^.	Harmonization with international standards (e.g., ISO 20387); implementation of SOPs, internal audits, and continuous quality monitoring^51,103^
Data Governance & Interoperability	Heterogeneous data models and poor interoperability between biobanks and databases^50^.	.Implementation of FAIR principles; adoption of standardized metadata (e.g., MIABIS); development of shared catalogues and interoperable APIs^104–106^
Ethical, Legal, and Social Issues (ELSIs)	.Uneven and incomplete biobanking regulations; inconsistent material transfer and ownership provisions^48,50,64^	Harmonization of national and regional frameworks; clear MTAs and governance models^57^; regional support through H3Africa and BCNet^51^.
	.Challenges in informed consent, particularly in rural and low-literacy settings; ethics review processes not tailored to biobanks^50,63^	Culturally adapted consent tools; community leader involvement as operationalized in co-created, context-sensitive consent frameworks developed for SSA communities^107^; establishment of biobank-specific ethics review boards^108,109^
	.Mistrust toward researchers, fears of exploitation, and unequal North-South collaborations (“dependency trap”)^52,63^	Transparent community engagement and fair benefit-sharing as demonstrated by the AWI-Gen model in Navrongo, Ghana^51^, and local analytic capacity building to ensure equitable partnerships^52^.
	.Tension between individual privacy and community-oriented ethics under Ubuntu philosophy (“I am, because we are”)^48,67^	Integration of Ubuntu principles in data governance; emphasis on communal trust, shared responsibility, and confidentiality^48,67^. as operationalized for example in the TRUCE model, an eight-step Ubuntu-inspired community engagement framework developed for genomic biobanking in South Africa^68^.
Human Resource and Funding	.Shortage of skilled personnel and limited mentoring capacity; researcher migration to HICs^52,70^	Structured training programs in genomics and bioinformatics^51,110^; regional centers of excellence; retention and career-development strategies^52^.
	.Overreliance on short-term, foreign-funded projects; low domestic R&D investment and limited private-sector involvement^52,74^	Diversified and sustainable funding portfolios; national investment in science; inclusion of cost-recovery mechanisms and service fees^52^
Visibility	Limited visibility and utilization of biobank resources by local researchers^111^.	Targeted dissemination through workshops, training sessions, and accessible biobank directories, as demonstrated by structured dissemination strategies developed within the BRoTHER-Biobank Network^112^.

### Infrastructural and logistical challenges

African biobanks often operate in resource-limited environments, causing significant infrastructural limitations^57,58^. Storage capacity and backup systems vary widely between African countries, leading to inconsistent preservation standards. Many sites lack adequate −80 °C freezer capacity for long-term storage and therefore depend on temporary −30 °C units or external facilities. In this context, secure and timely shipment is critical to maintain sample quality. However, the logistics of specimen transport are difficult, as additional export permits, cold-chain maintenance with dry ice, and courier delays can disrupt sample integrity^59^.

Although electricity supply and temperature monitoring systems are essential for preserving sample quality, power instability remains challenging^50^. In Nigeria, for example, several facilities reported the use of alternative energy sources to secure biobank operations. Akinyemi et al. report the use of solar-powered hybrid inverter-battery systems to maintain −80 °C freezers during power interruptions^57^.

A further challenge is the interoperability of equipment and digital systems. Many institutions rely on heterogeneous collections of outdated instruments acquired through different projects and donors^50,52^. Additionally, sequencing capacity remains insufficient in many laboratories, with outdated or low-throughput instruments restricting the generation and analysis of high-quality genomic data^52^. Limited maintenance capacity and a reliance on externally based engineers further constrain a reliable biobanking workflow^52^. Furthermore, information technology infrastructure is still at an early stage of development. In several biobanking locations, data are managed with basic spreadsheets instead of suitable laboratory management systems^50^. However, open-source software, such as the Africa-developed *Baobab Laboratory Information Management System*, addresses these limitations by providing standardized sample tracking, freezer monitoring, and harmonization^60^.

The expanding field of genomics and precision medicine produces large data volumes that demand advanced computational infrastructure. In this context, limited internet connectivity continues to hinder collaboration, data exchange, and the use of remote computing environments^52,61^.

### Challenges in ethical, legal, and social issues (ELSIs) in African biobanking

Biobanking activities must ensure the protection of participants’ rights and confidentiality, supported by explicit procedures for informed consent and clearly defined ELSIs^50,62^. Persistent challenges are closely linked to global power imbalances and the unequal distribution of research capacities between African countries and HICs^63^. Over the past decades, regulatory frameworks for biobanking in SSA have progressed, but their development is uneven across the continent. Several countries, such as South Africa, Kenya, Uganda, and Nigeria, have introduced specific acts or guidelines, while others are still in early or fragmented stages of regulation. These regional differences reflect disparities in governance capacity, digital infrastructure, and political stability^48,64^. Even where regulatory acts exist, provisions on material transfer remain incomplete or inconsistently applied, leaving uncertainties about ownership and sample export^64,65^. According to Mohammadzadeh et al.’s recent analysis, the identification of national regulatory acts is often difficult due to limited accessibility and inconsistent publication practices. Several frameworks exist only in draft or preparatory form, and information on their implementation is frequently incomplete or unavailable^48^. Ethical review structures are still primarily designed for individual research projects. As a result, biobank-specific ELSIs such as long-term storage, secondary use, and data sharing are not systematically reviewed. This creates a regulatory gap, as existing frameworks do not yet address the distinct ethical dimensions of sample and data stewardship^50^.

Notably, decades of infectious disease research and H3Africa-associated genomic research have generated substantial ELSI expertise in SSA, including context-adapted approaches to informed consent, community engagement, and sample governance in resource-constrained settings^48,66^. However, this knowledge has only been partially translated into oncology biobanking and cancer genomics, representing an underutilized resource for precision oncology in the region.

Informed consent remains a critical process, particularly in rural areas where poverty, limited literacy, and collective decision-making by local leaders influence participation^63^. Data protection in African biobanking should be interpreted within the ethical framework of *Ubuntu* (“I am, because we are.”), which regards privacy as a shared social responsibility rather than an individual possession. Personal data are treated with respect as part of maintaining trust and mutual care within the community. This understanding requires open explanation, recognition of communal interests, and consistent protection of confidentiality during all stages of data use^48,67^. Practical operationalizations of Ubuntu-informed governance have begun to emerge. The TRUCE model developed at Tygerberg Hospital in South Africa translates Ubuntu principles into an eight-step community engagement framework for genomic biobanking, emphasizing co-creation of consent processes, benefit-sharing, and community stewardship of biospecimens^68^. Despite these emerging frameworks, mistrust toward researchers persists, including fears of misuse, ritual exploitation, or commercial use of samples without consent^63^. Furthermore, there exist concerns that SSA could continue to serve mainly as a source of biospecimens for HICs, supporting the so-called dependency trap. Historical patterns of exporting samples for offshore analysis with limited local investment have positioned African teams as collectors rather than equal investigators^52,69^. Building equitable African biobanking relies on strengthening local governance, harmonizing regulations, taking culturally grounded practices into account, and developing research capacity to ensure trust, ethical stewardship, and fair international collaboration.

### Human resource and funding challenges

A chronic shortage of qualified personnel impedes the development of biobanking capacity and genomic research across the African continent. Many highly trained scientists migrate to HICs to find stable career prospects and better research environments, which further weakens local research capacity^70,71^. Furthermore, there exists a shortage of senior researchers who may mentor students and coordinate training in genomics and bioinformatics. This situation sustains reliance on external expertise and reinforces existing structural skill gaps^5,61^. Similarly, there is a severe deficit of professionals in bioinformatics, machine learning, and artificial intelligence who can manage, analyze, and interpret complex genomic datasets^52^. In this context, targeted training initiatives led by African institutions, such as the African Organization for Research and Training in Cancer (AORTIC), represent important steps toward strengthening locally led oncology research capacity across the continent^72,73^. However, funding conditions further aggravate these challenges. Research in LMICs often depends on short-term external grants, primarily from HICs, which limits sustainability and long-term planning^74^. Therefore, biobanks are commonly established as project-specific platforms and often result in disease-specific biospecimen collections that rarely develop into a lasting research infrastructure beyond the grant period^50^. Domestic investment in research is extremely low. In 2014, African countries spent on average only 0.4% of their GDP on research and development. In contrast to many HICs, the African private sector also lacks the capacity to compensate for public underfunding^52^. While pharmaceutical collaborations are an established source of biobank funding in HICs^75^, this mechanism remains largely absent in SSA^52^.

As a result, most biobanks and research programs operate without a sustainable financial base and are highly exposed to shifts in donor priorities^52^. This dependence on cyclical external support leads to the so-called “dependency trap,” where infrastructure and human capacity depend on foreign agendas and funding rhythms, undermining long-term stability^52^. Recent political developments illustrate these risks clearly. The withdrawal of USAID and other major donors from global health programs has caused abrupt funding gaps in several countries, revealing the fragility of aid-dependent health systems^76^. Despite profound implications for vulnerable populations, some researchers interpret this disruption as a potential turning point. According to Sarker et al., this situation offers an opportunity to strengthen national health systems through domestic financing, greater efficiency, and stronger political accountability^77^. Addressing these challenges from a biobank perspective requires sustainable financing and dissemination strategies. Particularly, young biobanks benefit from diversified funding portfolios to reduce vulnerability and maintain operational sustainability^78^. Introducing cost-recovery mechanisms, such as service fees, may help achieve financial independence and long-term sustainability^79^.

## From challenges to action: building a locally anchored and sustainable biobank ecosystem

The sustainability of biobank ecosystems in SSA depends on the development of a domestic research culture in which African scientists define the research questions, lead the analyses, and retain ownership of the data. Firstly, when research agendas are shaped by local priorities, such as population-specific cancer prevention strategies, risk stratification models, and biomarker discovery, they generate scientific value that is directly translatable into clinical benefit for local populations. Secondly, this creates a momentum that makes research careers on the continent attractive, thereby reducing the structural incentive for the so-called *brain drain*. Thirdly, conducting analyses locally is furthermore not only ethically preferable but also economically rational. Based on our own collaborative experience, certain cost components, particularly personnel costs, are lower in SSA than in HICs, although this may be equalized by higher costs for imported reagents and infrastructure constraints^80^. However, strengthening local capacity has the potential to improve efficiency over time and reduce dependence on external analysis pipelines. To achieve this, sustainable biomedical research requires public commitment from local stakeholders. African governments should recognize biobanking and precision oncology as domestic health priorities and invest accordingly. Within the broader region, Egypt offers examples of locally anchored investment in cancer. The government has made cancer a national health priority through the publicly funded *Presidential Initiative on Women’s Health*^81^. In addition, several disease-based biobanks, including an oncology biobank at the National Cancer Institute, Cairo University, have been established and linked through a national biobank network^82^. Until now, such locally anchored models remain rare across SSA.

At the same time, external partnerships must respect local research autonomy to prevent helicopter research. Although South-South collaboration is frequently emphasized at the political level, infrastructural fragmentation, limited interoperability, and uneven regulatory harmonization continue to constrain its implementation. This underlines that regional integration is as critical as international partnerships. The Mali-Germany biobanking network illustrates one potential model for this transition: a long-term, equitable collaboration that prioritizes mutual learning, context-specific adaptation, and capacity building of local biobank personnel rather than replicating external models^83^.

## Limitations

This review has limitations inherent to its narrative design. As a narrative review, the literature search was targeted, and selection bias cannot be excluded. For emerging and rapidly evolving topics such as private-sector biobanking initiatives, grey literature including journalistic sources was consulted where peer-reviewed evidence was limited.

## Conclusion

As many HICs turn inward, SSA stands at a crossroad between persisting challenges and new opportunities in public health. The burden of infectious diseases is gradually declining, while life expectancy rises and cancer incidence on the continent is projected to increase. In the context of Africa’s immense genetic diversity, investment in research and precision medicine holds promise. Importantly, genetic diversity exists not only between African and non-African populations but also among populations within the African continent. Therefore, precision medicine approaches should rely on population-specific data and avoid assumptions of genetic uniformity across Africa. This literature review highlights how local biobanks, such as the ARGO colorectal cancer biobank in Nigeria, already contribute to precision oncology. To ensure that SSA becomes not only a recipient but an active player in global precision oncology, several foundational elements are required: clear regulatory frameworks, sustainable biomedical infrastructure that enables local analyses, long-term funding mechanisms, local expertise, and equitable international partnerships. Whether *Precision Oncology Without Borders* becomes an equitable reality will ultimately depend on the willingness of African governments, international funders, and research institutions to prioritize local research over hierarchical models of collaboration.

## Data Availability

No datasets were generated or analysed during the current study.
